# Trans-inhibition of HIV-1 by a long hairpin RNA expressed within the viral genome

**DOI:** 10.1186/1742-4690-4-15

**Published:** 2007-03-01

**Authors:** Pavlina Konstantinova, Olivier ter Brake, Joost Haasnoot, Peter de Haan, Ben Berkhout

**Affiliations:** 1 Laboratory of Experimental Virology, Department of Medical Microbiology, Center for Infection and Immunity Amsterdam (CINIMA), Academic Medical Center, University of Amsterdam, Meibergdreef 15, 1105 AZ Amsterdam, The Netherlands; 2 Viruvation B. V. Wassenaarseweg 72, 2333 AL Leiden, The Netherlands

## Abstract

**Background:**

Human immunodeficiency virus type 1 (HIV-1) can be inhibited by means of RNA silencing or interference (RNAi) using synthetic short interfering RNAs (siRNAs) or gene constructs encoding short hairpin RNAs (shRNAs) or long hairpin RNAs (lhRNAs). The use of siRNA and shRNA as antiviral therapeutic is limited because of the emergence of viral escape mutants. This problem is theoretically prevented by intracellular expression of lhRNAs generating multiple siRNAs that target the virus simultaneously, thus reducing the chance of viral escape. However, gene constructs encoding lhRNA molecules face problems with delivery to the right cells in an infected individual. In order to solve this problem, we constructed an HIV-1 variant with a 300 bp long hairpin structure in the 3' part of the genome corresponding to the Nef gene (HIV-lhNef).

**Results:**

Intriguingly, HIV-lhNef potently inhibited wild-type HIV-1 production in trans. However, HIV-lhNef demonstrated a severe production and replication defect, which we were able to solve by selecting spontaneous virus variants with truncated hairpin structures. Although these escape variants lost the ability to trans-inhibit HIV-1, they effectively outgrew the wild-type virus in competition experiments in SupT1 cells.

**Conclusion:**

Expression of the lhNef hairpin within the HIV-1 genome results in potent trans-inhibition of wild-type HIV-1. Although the mechanism of trans-inhibition is currently unknown, it remains of interest to study the molecular details because the observed effect is extremely potent. This may have implications for the development of virus strains to be used as live-attenuated virus vaccines.

## Background

RNA interference (RNAi) has been used to inhibit the replication of a wide range of viruses including the human immunodeficiency virus type 1 (HIV-1), hepatitis C virus (HCV), hepatitis B virus (HBV), dengue virus, poliovirus, influenza virus A, coronaviruses, herpesviruses, and picornaviruses [1,2]. Due to its sequence specificity, RNAi is a potentially selective method for intracellular immunization against HIV-1 infection. RNAi-mediated suppression of HIV-1 replication has been accomplished by synthetic small interfering RNAs (siRNAs) in a transient manner [3-6] and by shRNA expression vectors in stably transfected cells [7-9]. Despite potent inhibition, the use of both approaches as therapeutic antiviral is limited because of the rapid emergence of HIV-1 escape mutants [9-11]. Strategies to reduce the chance of viral escape include the simultaneous use of multiple siRNAs [12,13], the intracellular expression of a second generation of escape-anticipating shRNAs [14], or microRNA-based double-stranded RNAs (miRNAs), which do not require perfect sequence complementarity for inhibition [15,16].

An alternative method to inhibit HIV-1 is the use of gene constructs encoding HIV-1-specific long hairpin RNAs (lhRNAs, transcripts folding an extended hairpin structure) or long double-stranded RNAs (dsRNAs, two complementary transcripts that form an extended duplex). These molecules should yield multiple effective siRNAs upon intracellular processing [6,17,18]. However, lhRNA approaches raise concerns about induction of the dsRNA-triggered interferon (IFN) response. Others and we have shown that endogenously expressed lhRNA and dsRNA can inhibit HIV-1 production without induction of the innate antiviral response [18-21]. In fact, most reports on IFN induction by long dsRNAs in mammalian cells are based on transfection of cells with in vitro synthesized dsRNAs [22,23]. Apparently, endogenously produced dsRNA is less active than exogenous dsRNA in inducing the IFN response.

Several antiviral approaches using extended lhRNA and long dsRNA molecules have been reported in plant and insect cells that lack the innate antiviral IFN response. Transient expression of DNA constructs encoding virus-specific dsRNA in plant protoplasts or insect cells partially protects the cells from infection by the homologous virus [23,24]. Stable expression of such constructs renders the cells resistant to infection [25,26]. lhRNA can inhibit HIV-1 production under certain conditions, without induction of the IFN response [6,17,18]. Ideally, a single lhRNA should generate multiple effective siRNAs upon intracellular processing, providing more durable inhibition of HIV-1 than a single shRNA. An additional advantage of lhRNA inhibitors is that it does not require pre-determination of the optimal shRNAs and corresponding HIV-1 target sequences because multiple effective siRNAs will be produced. A potential disadvantage of the use of lhRNA as therapeutic is that the generation of multiple siRNAs will be more likely to cause off-target effects.

We have previously reported strong inhibition of HIV-1 production using gene constructs encoding HIV-specific lhRNAs and dsRNA in transient transfection assays [18]. An alternative for stable expression of shRNAs is a conditionally replicating HIV-1-based virus, which was previously used by us to deliver an antiviral shRNA cassette into HIV-1 susceptible target cells [27]. In the current study, we constructed the HIV-lhNef variant, which contains a 300 bp extended hairpin structure at the 3' genome position of the Nef gene of the otherwise wild-type HIV-1. We tested this HIV-lhNef for its capacity to inhibit the production of wild-type virus. Intriguingly, HIV-lhNef potently inhibited wild-type HIV-1 production in trans. However, HIV-lhNef demonstrated a severe production and replication defect, which we were able to solve by selecting spontaneous, escape viruses.

## Results

### The HIV-lhNef variants

**Figure 1:**
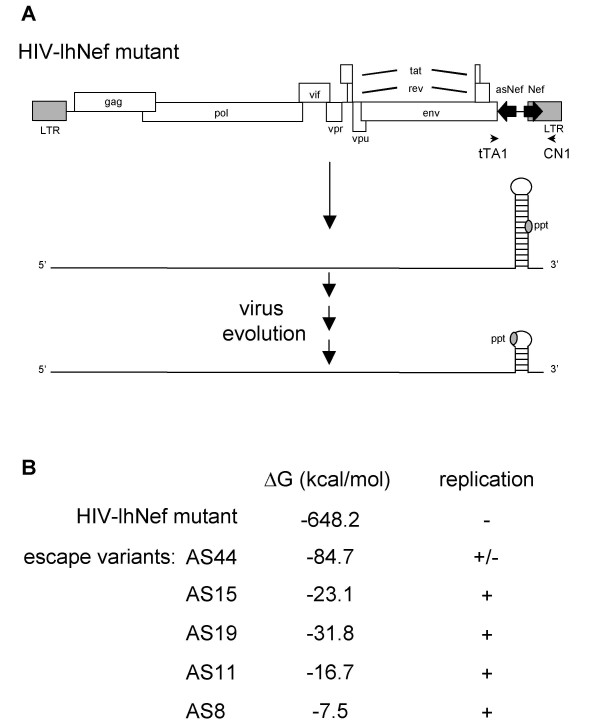
The HIV-lhNef RNA genome encodes an extended hairpin that is truncated by virus evolution. (A) The proviral DNA genome, highlighting the insertion of the antisense asNef fragment (original Nef coordinates +8544 to +8844). The primers tTA1 and CN1 used to amplify the inverted repeat region are indicated. The inverted repeat (thick arrows) results in the formation of a 300 bp hairpin structure in the RNA genome of HIV-lhNef (middle panel). This hairpin encompasses the polypurine tract (ppt). The truncated hairpin structure that emerged by virus evolution is shown in the lower panel. (B) HIV-lhNef escape variants were previously described in detail [28]. The name AS (antisense) reflects the number of basepairs in the remaining RNA hairpin. ΔG is the thermodynamic stability of the remaining perfectly basepaired stem segment. The replication column shows the replication capacity of the viruses.

We previously described the construction of the HIV-lhNef virus variant [28], which contains a 300 bp extended hairpin structure (Fig. 1A). The hairpin structure is present in the full-length genomic RNA and all spliced mRNAs. This structure induced a severe virus production and replication defect. Similarly, we were not able to obtain stable expression of the lhRNA inhibitor from a lentiviral vector (results not shown), probably due to problems in reverse transcription of the excessively stable hairpin structure [29]. The presence of the lhNef insert resulted in a dramatic drop of the viral transduction titer, possibly also due to self-targeting of Nef/LTR sequences in the vector genome. Unlike the non-replicating lentiviral vector, the HIV-lhNef virus could generate spontaneous variants by evolution. Indeed, replicating virus variants could be selected with a severely truncated lhNef hairpin structure [28]. These escape variants are listed in Figure 1B, with the number of basepairs in the remaining hairpin structure in their name. For instance, AS44 has 44 remaining basepairs, producing a hairpin of intermediate stability (ΔG = - 84.7 kcal/mol). We now set out to test these variants in further detail, e.g. for their ability to inhibit wild-type HIV-1 in trans.

### Trans-inhibition of wild-type HIV-1

**Figure 2:**
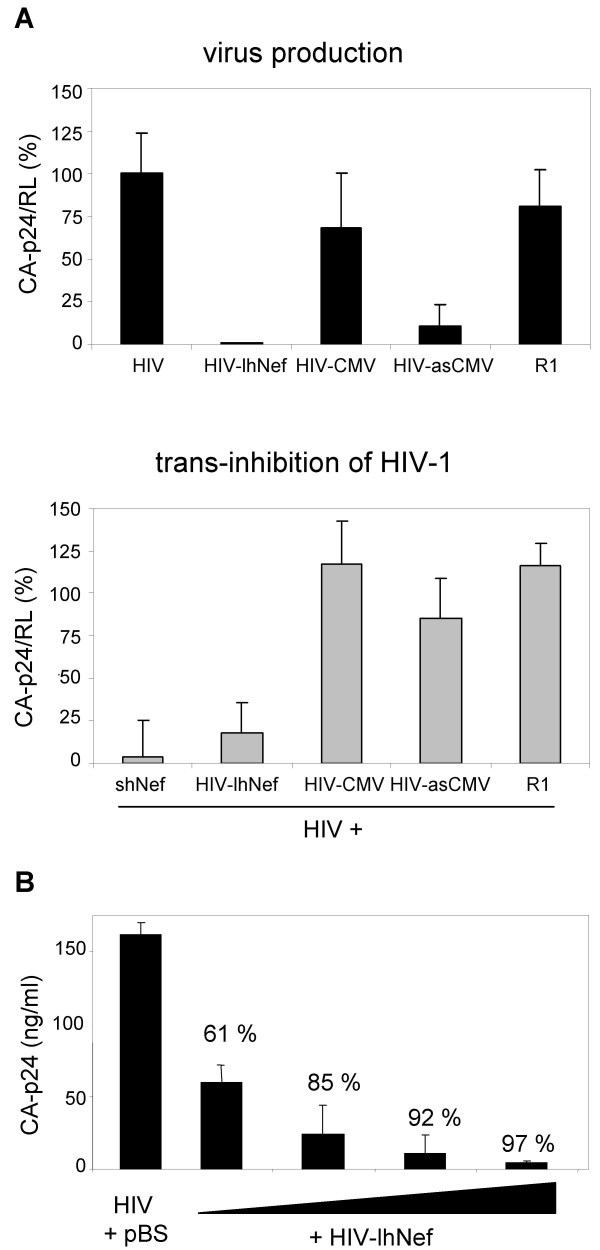
Trans-inhibition of HIV-1 by HIV-lhNef. (A) HEK239T cells were co-transfected with 150 ng of the HIV-1 molecular clone LAI, HIV-lhNef, HIV-CMV, HIV-asCMV or R1 and 150 ng pBluescript (Promega) (upper panel). In the lower panel 150 ng HIV was co-transfected with 150 ng pSuper-shNef, HIV-lhNef, HIV-CMV, HIV-asCMV or R1. 1 ng pRL plasmid, expressing Renilla luciferase from the CMV promoter was added as an internal control for cell viability and transfection efficiency. Transfections were performed with lipofectamine-2000 and 1.5 × 10^5 ^cells. Virus production was measured in the culture supernatant 2 days after transfection by CA-p24 ELISA and Renilla expression was measured with the Renilla luciferase assay system (Promega). We plotted the relative percentage of CA-p24/RL, with the HIV + pBluescript transfection set at 100%. Error bars represent the standard deviation from quadruple transfections in three independent experiments. (B) Titration of the HIV-lhNef inhibitor. 500 ng HIV-1 was co-transfected with increasing amounts (0-75-125-250-500 ng) of HIV-lhNef. The total DNA concentration was kept constant by adding pBluescript. Virus production was measured in the culture supernatant 2 days after transfection. Error bars represent the standard deviation in three replicates. This is a representative figure from three transfection experiments with similar results.

HEK293T cells were transfected with the wild type HIV-1 construct or HIV-lhNef. We measured CA-p24 in the culture supernatant as a measure of virus production 2 days post-transfection. Whereas wild-type HIV-1 produced high CA-p24 levels, no virus production was detected for HIV-lhNef (Fig. 2A, upper panel). This loss of virus production is due to the presence of the lhNef hairpin because no such effect was scored for several control constructs: HIV-1 with a CMV insertion in sense and antisense orientation (HIV-CMV and HIV-asCMV) and the Nef-deleted HIV-1 construct R1 (Fig. 2A). Notably, HIV-asCMV did not produce high CA-p24 values probably due to promoter interference from the strong CMV promoter. We next co-transfected equal amounts of wild-type HIV-1 with HIV-lhNef, HIV-CMV, HIV-asCMV or R1 (Fig. 2A, lower panel). Interestingly, only HIV-lhNef was able to potently inhibit wild-type HIV-1 production in trans. The level of inhibition is comparable to that obtained in a co-transfection with the highly effective shNef inhibitor [9], which we used as a positive control. We next titrated the HIV-lhNef construct to test if the inhibitory effect is concentration-dependent (Fig. 2B). We measured 92% inhibition of wild-type virus production when mixed 2:1 with the HIV-lhNef inhibitory construct. Even at a 7:1 ratio, HIV-1 production was reduced by 61%. The mechanism of this potent inhibition is currently unknown. Although HIV-lhNef may be a potently interfering construct, a major problem is that it does not replicate.

### Replication properties of the escape viruses

**Figure 3:**
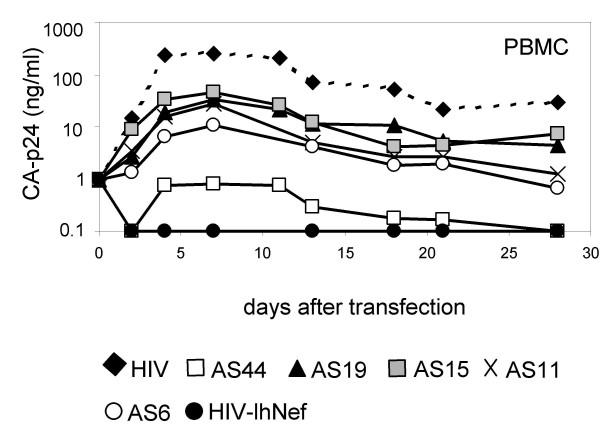
Virus replication of HIV-lhNef and the escape variants in PBMC. Cells (5 × 10^6^) were transfected with 10 μg of the proviral DNA constructs and CA-p24 production was measured in the culture supernatant at several days post-transfection for up to 4 weeks. Half of the culture medium was replaced with fresh complete RPMI medium with IL-2 every 4 days and freshly activated PBMC (2 × 10^6^) were added at every 2^nd ^addition. The experiment has been repeated five times for AS19 and two times for the other variants.

We studied the replication potential of HIV-lhNef and the AS escape variants in PBMC (Fig. 3). As a positive control, the Nef-positive wild-type HIV-1 construct was used. As shown previously, HIV-lhNef did not replicate to detectable levels. The escape variant AS44, with the intermediate length hairpin, replicated only marginally. All other AS variants replicated efficiently (results are summarized in Figure 1B), although these variants reached maximal CA-p24 values at least 1 log lower than that of wild-type HIV-1. This phenotypic difference can be explained by the Nef-minus genotype of these viral strains as the accessory Nef protein contributes to efficient virus replication in primary cells [30,31].

**Figure 4:**
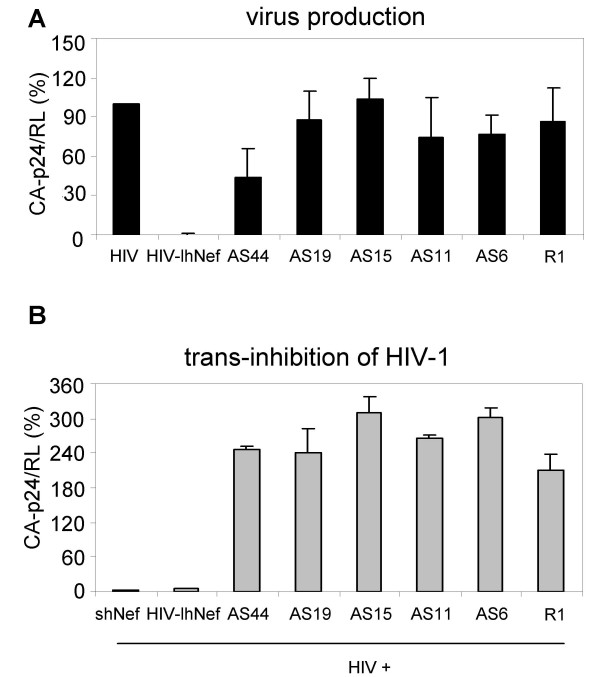
Virus production of the HIV-lhNef escape variants and trans-inhibition of HIV-1. (A) Virus production in HEK293T cells. Cells were transfected with 150 ng pBluescript and 150 ng of the indicated constructs. 1 ng pRL plasmid was added as an internal control. Transfections were performed with lipofectamine-2000 and 1.5 × 10^5 ^cells. Virus production was measured in the culture supernatant 2 days after transfection by CA-p24 ELISA and RL expression was measured with the Renilla luciferase assay system (Promega). We plotted the relative percentage of CA-p24/RL, with the transfection HIV + pBluescript set at 100%. Error bars represent the standard deviation from quadruple transfections in two independent experiments. (B) Trans-inhibition of HIV-1 production by the AS escape mutants. HEK293T cells were co-transfected with 150 ng HIV-1 and 150 ng pSuper-shNef, HIV-lhNef, the AS escape mutants or R1. CA-p24 production was measured in the culture supernatant at two days post-transfection. Error bars represent the standard deviation from quadruple transfections in two independent experiments.

We next transfected the plasmid constructs encoding wild-type HIV-1, HIV-lhNef and the AS escape variants in HEK293T cells. These cells do not support HIV-1 replication, but produce virus particles upon DNA transfection. Virus production was measured by CA-p24 ELISA in the culture supernatant at three days post-transfection. Unlike the original HIV-lhNef mutant, which demonstrated a severe CA-p24 production defect, all AS variants efficiently produced virus (Fig. 4A). This result demonstrates that truncation of the lhNef hairpin overcomes the production and replication defect. We next tested if the AS variants are able to inhibit wild-type HIV-1 in trans in the co-transfection assay. Compared to the effective HIV-lhNef inhibitor, all AS escape variants had lost the ability to actively inhibit wild-type virus production (Fig. 4B). Even the poorly replicating AS44 variant lost the capacity to inhibit HIV-1 in trans. Thus, the capacity of HIV-lhNef to inhibit HIV-1 correlates with its replication defect.

### Virus competition between HIV-1 and the HIV-lhNef escape variants

As a more sensitive assay for possible trans-inhibition, we tested the individual AS variants in a direct competition with wild-type HIV-1 in PBMC. Cells were infected with an equimolar mixture of the two viruses (based on CA-p24) that were produced in HEK293T cells, and virus was passaged to fresh cells at the peak of infection. Virus replication was monitored by CA-p24 ELISA and visual inspection for syncytia. Cellular DNA was extracted at several times post-infection and the proviral Nef region was PCR amplified with primers tTA1 and CN1 (see Fig. 1). Since the PCR products will differ in size for wild-type HIV-1 and each AS variant, both competitors can be detected in the same sample by subsequent agarose gel electrophoresis of the DNA fragments. The outgrowth of a particular virus was verified by cloning and sequencing of the PCR products from the last time-point sample (results not shown).

**Figure 5:**
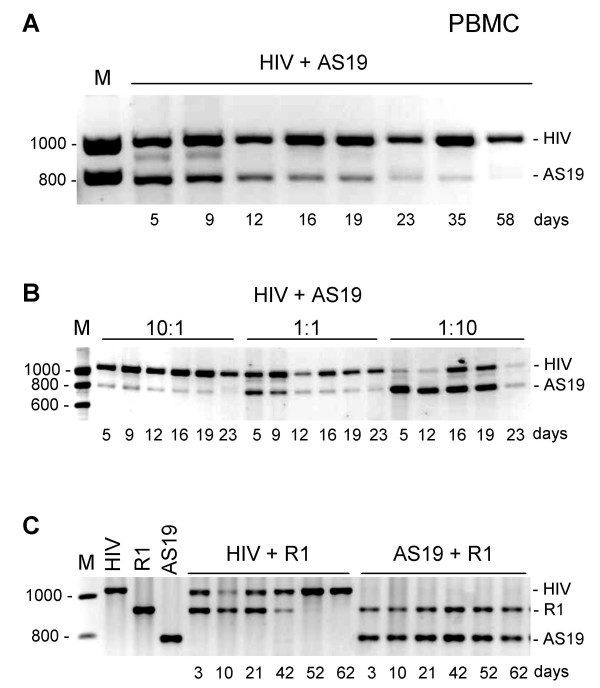
Virus competition experiments in PBMC. (A) Two viruses were mixed as indicated on top of the panels, usually in a 1:1 ratio (unless indicated otherwise, see panel B). The composition of the virus mixture was followed by PCR across the Nef region with primers tTA1 and CN1 (see Fig. 1A). The experiment has been repeated 5 times for AS19. Appropriate markers and molecular weight standard (M) are indicated. The identity of the respective PCR fragments is indicated on the right. (B) Competition of HIV-1 and AS19 mixed in three ratios: 10:1, 1:1, 1:10. (C) Competition between the R1 variant and either HIV-1 or AS19. R1 is a 106 nt Nef deletion mutant. The experiment has been repeated twice, with similar results.

Wild-type HIV-1 effectively outcompeted all AS mutants in PBMC. An example of the competition between HIV-1 and the AS19 variant is provided in Figure 5A. Both viruses are detected in the culture at day 5, but we observed a gradual increase in the intensity of the larger PCR product. A single PCR product was observed at day 58, indicating that HIV-1 predominated the culture and thus effectively outcompeted AS19. The same result was obtained when HIV-1 and AS19 were mixed in a 1:10 ratio (Fig. 5B). The competition results for all AS variants are summarized in Table 1 and shown in Additional file 1. The AS variants are likely to be less replication competent than wild-type HIV-1 due to the Nef-minus genotype, but the remnant hairpin structure may also impose a negative effect on the replication capacity. We therefore included the unrelated Nef-minus mutant R1 with a 106 nt deletion in Nef, but without a hairpin structure [9]. R1 produces CA-p24 levels comparable to wild-type HIV-1 and cannot inhibit viral production in trans (Fig. 2 and 4). We performed competition experiments between HIV-1 and R1, but also with AS19 and R1. HIV-1 also outcompetes the alternative Nef-minus R1 mutant (Fig. 5C). AS19 and R1 co-existed in the culture up to 62 days post-infection (Fig. 5C), indicating that both Nef-minus viruses have very similar replication fitness.

**Table 1:** Pairwise virus competition experiments

Virus mix ^a^	PBMC winner	SupT1 winner
HIV/AS44	HIV (4)^b^	HIV (4)
HIV/AS19	HIV (58)	AS19 (12)
HIV/AS15	HIV (23)	AS15 (23)
HIV/AS11	HIV (23)	AS11 (33)
HIV/AS6	HIV (11)	AS6 (16)
HIV/R1	HIV (52)	HIV/R1 (>62)
AS19/R1	AS19/R1 (>62)	AS19/R1 (>62)

^a ^the same virus mix was tested on PBMC and SupT1 cells

^b ^the day of outgrowth (>90%) is indicated between brackets; the experiment has been repeated five times for AS19 and two times for the other variants

**Figure 6:**
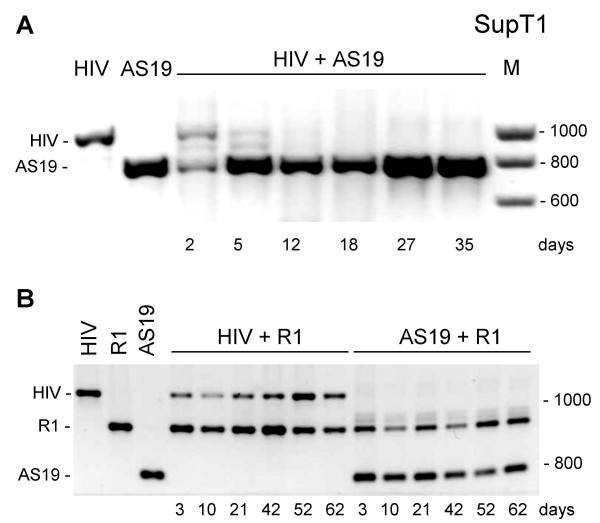
Virus competition experiments in the SupT1 T cell line. See legend to Fig. 5 for details. (A) Competition between HIV-1 and AS19. (B) Competition between the R1 variant and either HIV-1 or AS19.

We repeated the competition experiments in the SupT1 T cell line. Because the Nef protein has no impact on viral replication fitness in T cell lines [30], this system may allow a more sensitive screen for trans-inhibition of wild-type HIV-1 by the hairpin-containing AS variants. In fact, all AS mutants outcompeted wild-type HIV-1, as illustrated for the AS19 variant (Fig. 6A). The results for all AS variants are summarized in Table 1 and shown in Additional files 1, 2 and 3. Intriguingly, variant R1 co-existed with the wild-type virus for 62 days (Fig. 6B). This result confirms that the Nef function is not important in T cell lines. However, because the AS variants were able to outcompete HIV-1, it raises the interesting possibility of hairpin-mediated (trans) inhibition by these AS variants. Perhaps even more surprisingly, AS19 and R1 co-existed for the length of the competition experiment. We speculate that the deletion mutant R1 may lack target sequences for trans-inhibition by AS19.

### Mechanism of trans-inhibition by HIV-lhNef and the AS variants

**Figure 7:**
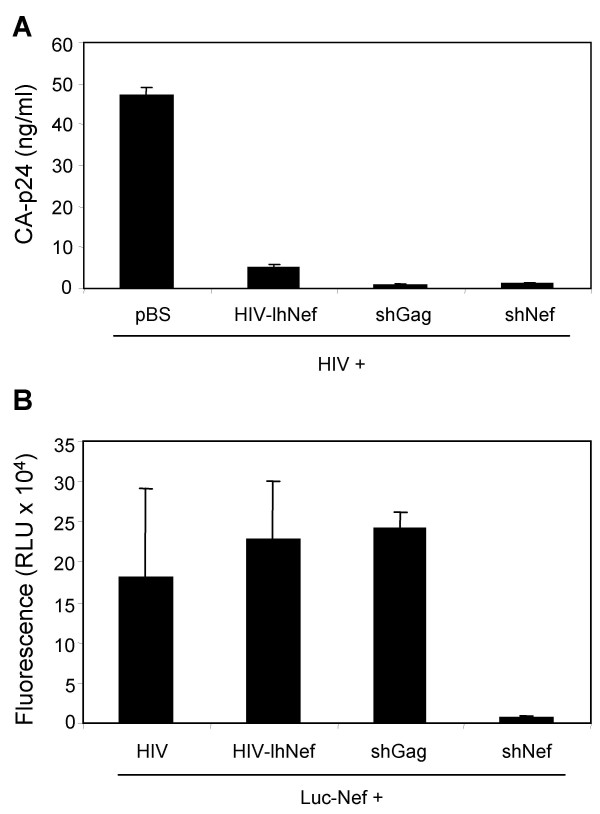
Test of sequence-specificity of the inhibition by HIV-lhNef. (A) Trans-inhibition of HIV-1 production by HIV-lhNef and the RNAi-inducing shGag or shNef constructs. HEK293T cells were co-transfected with 100 ng HIV-1 and 100 ng pBluescript, 100 ng HIV-lhNef, shGag or shNef. CA-p24 production was measured in the culture supernatant at two days post-transfection. Error bars represent the standard deviation in two independent experiments. (B) Inhibition of the Luc-Nef reporter. HEK 239T cells were co-transfected with 100 ng of Luc-Nef and 100 ng HIV-1, HIV-lhNef, shGag or shNef. Error bars represent the standard deviation in two independent experiments.

We have previously described strong inhibition of HIV-1 by RNAi-inducing expression vectors encoding shRNA or lhRNA molecules directed against viral genes [9,13,14,18]. One possibility is that the mechanism of HIV-1 inhibition by HIV-lhNef or the AS mutants could be RNAi-related. One of the hallmarks of RNAi is its sequence-specificity. We therefore tested if HIV-lhNef could inhibit the Luc-Nef reporter, in which a 250 nt Nef target sequence was placed downstream of the *Photinus *luciferase gene [11]. As a positive control, we showed that HIV-lhNef induced a dramatic decrease of wild-type HIV-1 production in trans, comparable in efficiency with the highly effective RNAi-inducers shGag and shNef [13] (Fig. 7A). However, no sequence-specific inhibition of the Luc-Nef reporter was obtained with HIV-lhNef (Fig. 7B). The shGag molecule serves as a negative control in this experiment, and shNef as a potent positive control. In these transient transfection experiments a Renilla luciferase expression plasmid was included, which provides a control for transfection efficiency and possible aspecific effects. Renilla luciferase expression was similar in all transfections (results not shown), indicating that HIV-lhNef mediated inhibition of HIV-1 is specific and not due to non-specific effects, e.g. due to IFN induction by dsRNA.

## Conclusion

In this study, we show that HIV-1 expressing an excessively stable 300 bp lhNef hairpin potently inhibits wild-type HIV-1 in trans. However, HIV-lhNef did not replicate to detectable levels in HIV-1 target cells, probably because steps of its replication cycle are affected by the hairpin insertion, e.g. RNA splicing, RNA nuclear export or mRNA translation. Moreover, HIV-lhNef may cause degradation of its own mRNA due to processing by the RNAi machinery. We postulate that the lhNef hairpin may induce an antiviral response against wild-type HIV-1 either through a sequence-specific RNAi mechanism or through aberrant HIV-1 transcripts lacking the 3' non-coding region and polyA tail that induce a potent HIV-1-specific RNA silencing response in cells. Tests with reporter constructs do not support a sequence-specific RNAi effect, although the effect induced by lhNef is HIV-1 specific. The mechanism of trans-inhibition is currently unknown, but it remains of interest to study the molecular details because the observed inhibition is extremely potent. We are currently dissecting the mechanism of inhibition by testing HIV-lhNef variants that lack important RNA signals (TAR, polyA, PAS, PBS, DIS, SD, psi).

Although HIV-lhNef is a potent inhibitor of wild-type HIV-1, its inability to replicate precluded long-term inhibition experiments. After prolonged culturing of HIV-lhNef, replicating variants emerged through recombination events that introduce large truncations in the lhNef hairpin structure and therefore deletions in the Nef gene. Interestingly, all AS variants inherit a part of the hairpin structure in their genome, there were no perfect deletions of the entire lhNef region. One could speculate that the remaining secondary RNA structures are actively selected because they render the viral genome less susceptible for degradation by the RNAi machinery [11].

Nef represents a pathogenicity factor that disorders adaptive immunity by down regulating CD4 and MHC-1 receptors, by inhibiting T-cell chemotaxis, and by inducing apoptosis in bystander T-cells, and hence plays a major role in the destruction of the host immune system [32,33]. It has been suggested that any therapeutic intervention aimed at either completely blocking or at least partially reducing the expression of Nef during HIV-1 infection would likely enhance the ability of the immune system to fight HIV infection [34]. Humans infected with Nef-defective HIV-1 strains show low viral loads and no or very slow disease progression and represent long-term non-progressors or long-term slow-progressors [31,35]. Moreover, macaques vaccinated with a SIV strain that only lacks Nef are better protected against superinfection than macaques vaccinated with a SIV strain lacking the three accessory genes Nef, Vpr and Vpx [36]. The cross-protection conferred by the attenuated SIV strains appears not to be based on stimulation of the adaptive immune system, but on other (unknown) mechanisms [37]. In this study, we demonstrate effective outgrowth of the Nef-minus AS escape variants in competitions with wild-type HIV-1. This result was obtained in the SupT1 T cell line, which does not provide a clear Nef-phenotype. We think that this outcompetition is related to the presence of the genomic hairpin structure, because no such effect was observed for the control Nef-deletion virus R1. These in vitro results may relate to the superior protection with Nef-deleted viruses in vivo.

Successful expression of a shRNA from a replicating viral vector has been shown for Rous sarcoma virus (RSV) in avian cells [38]. We previously described inhibition of HIV-1 replication by a conditional-live HIV-rtTA virus that expresses a shRNA against the Nef gene [27]. This approach is especially suitable for targeting cells that are susceptible to HIV-1 infection. However, HIV-1 escape variants will emerge rapidly under shNef pressure [9]. Expression of multiple shRNAs or a single lhRNA from such a vector could prevent viral escape because multiple precise mutations should occur. Use of a murine leukemia virus (MLV) can also be used for efficient and stable delivery of anti-HIV-1 shRNA [39]. However, MLV can replicate only in actively dividing cells, which limits its application as a therapeutic virus.

One ideal property of replicating HIV-1-based viral vectors is that they specifically target HIV-1 susceptible cells. The replication-competent AS escape variants lost the trans-inhibitory properties of HIV-lhNef, but effectively outcompeted wild-type HIV-1 in T cells. It is important to asses what is the minimum length of a hairpin that can mediate trans-inhibition of wild type HIV-1. We have previously shown that a 19 nt shRNA expressed from conditionally replicating HIV-1-based virus, can inhibit viral replication [27]. Any hairpin longer than that should in theory mediate trans-inhibition of HIV-1. Further research is needed to see if we can design constructs that are replication competent, yet remain a potent trans-inhibitor of HIV-1. For example, a lhRNA variant with G-U wobbles could be designed, which will destabilize the RNA structure and therefore stimulate viral replication. Such an approach using 90–100 bp lhRNA molecules has been suggested as a means for intracellular immunisation against HBV and HCV, without evoking non-specific IFN responses [19,40]. Such constructs could form the basis for an attenuated virus vaccine and anti-HIV therapeutic virus in one.

## Materials and methods

### DNA constructs and proviral DNA analysis

The full-length molecular HIV-1 clone LAI [41] (Accession number AF33819.3) was used to produce wild-type virus. HIV-lhNef has a 93 nt deletion in the 5' region of Nef and a 300 bp inverted repeat within the Nef gene (Fig. 1). The construction of the HIV-lhNef mutant and the generation of molecular clones of HIV-lhNef escape virus variants AS44, AS19, AS15, AS11 and AS8 has been described previously [28]. The HIV-1 mutant R1 has a 106 nt deletion in Nef (+8513 to +8619) and has been described previously [9]. HIV-CMV and HIV-asCMV were constructed by inserting the CMV promoter in sense or antisense orientation at position +8069 of the LAI genome. pSuper-shGag and pSuper-shNef with the H1 polymerase III promoter have been described previously [13]. Nucleotide numbers refer to the position on the genomic HIV-1 RNA transcript, with +1 being the capped G residue. Plasmid pGL3-Nef (Luc-Nef), in which 250 nt from the Nef gene was placed downstream of the *Photinus *luciferase reporter gene has been described previously [11].

For cellular DNA isolation, cells were pelleted for 4 min at 4000 rpm and solubilized in 150 μl lysis buffer (10 mM Tris-HCl pH 8.0, 1 mM EDTA, 0.5 % Tween 20) and 200 μg/ml proteinase K (Roche) at 56°C for 1 h and at 95°C for 10 min. Proviral DNA sequences were PCR amplified from 5 μl cellular lysate using the 5' Env primer tTA1-AD (+8269 to +8289, ACA GCC ATA GCA GTA GCT GAG) and 3' U5 primer CN1 (+9283 to +9253, GGT CTG AGG GAT CTC TAG TTA CCA GAG TC) (Fig. 1). PCR amplification was performed in a 50 μl reaction containing 1 × PCR amplification buffer (Invitrogen), 0.5 mM MgCl_2_, 25 pmol of each primer, 0.2 mM dNTPs and 2.5 units of AmpliTaq DNA polymerase (Perkin Elmer Applied Biosystem). The PCR program was as follows: 95°C for 5 min, 30 cycles of 30 sec at 94°C, 30 sec at 55°C, 30 sec at 72°C, and a final extension for 7 min at 72°C. The PCR products were separated on a 1% agarose gel stained with ethidium bromide and compared to a standard DNA size marker (Eurogentec). HIV-lhNef could not be amplified with this PCR due to the extended inverted repeat that may form a cruciform DNA structure. The RNA structures formed by HIV-lhNef and the AS escape variants were predicted with the Mfold program [42].

### Cells, DNA transfection and virus infection

Human embryonic kidney (HEK) 293T cells were grown as a monolayer in DMEM (Invitrogen) supplemented with 10% FCS, minimal essential medium nonessential amino acids, penicillin (100 U/ml) and streptomycin (100 μg/ml) at 37°C and 5% CO_2_. One day before transfection, cells were trypsinized, resuspended in DMEM and seeded in 24-well plates at a density of 1.5 × 10^5 ^cells per well. Cells were co-transfected with 100 – 500 ng DNA with Lipofectamine 2000 (Invitrogen) according to the manufacturer's instructions. 1 ng pRL plasmid (Promega), expressing Renilla luciferase (RL) from the CMV promoter, was added as an internal control for cell viability and transfection efficiency. In all transfection experiments we have controlled for the DNA input by adding pBluescript (Promega) to reach identical DNA concentrations.

Peripheral blood mononuclear cells (PBMCs) were isolated from buffy coats by standard Ficoll-Hypaque density centrifugation, activated with phytohemagglutinin (3 μg/ml), and cultured in complete RPMI 1640 medium (Invitrogen) with IL-2 (100 U/ml). Cells (5 × 10^6^) were transfected with 10 μg of the proviral DNA by electroporation in 250 μl RPMI with 20% FCS in 0.4-cm cuvettes at 250 V and 960 μF, and 1 × 10^6 ^fresh cells and 5 ml complete RPMI with IL-2 were added afterwards. PBMCs were infected with a fixed amount (5 ng CA-p24) of 293T-produced virus and maintained for up to 4 weeks. Half of the culture medium was replaced every 4 days by fresh complete RPMI 1640 medium with IL-2 and freshly activated PBMC (2 × 10^6^) were added at every 2^nd ^addition.

The human T cell line SupT1 was cultured in RPMI 1640 medium supplemented with 10% fetal calf serum (FCS) (Hybond), penicillin (100 U/ml) and streptomycin (100 μg/ml) at 37°C and 5% CO_2_. Cells were cultured in 25 cm^2 ^flasks and split 1 to 10 twice a week. Cells were infected with equal amounts (5 ng CA-p24) of 293T-produced virus. When HIV-induced cytopathic effects were observed, virus replication was maintained by passage of the cell-free culture supernatant onto uninfected SupT1 cells. At each passage, cell and supernatant samples were stored at -70°C. Virus spread was followed by CA-p24 ELISA on the culture supernatant as described previously [43] and Renilla expression was measured with the Renilla luciferase assay system (Promega).

## Supplementary Material

Additional File 1 Virus competition experiments in SupT1 and PBMC. The composition of the wild type HIV-1 and the AS escape mutants was followed by PCR across the Nef region with primers tTA1 and CN1 (see Fig. 1). Click here for file

Additional File 2 Graphic quantification of the relative viral abundance in SupT1 and PBMC. The density of the PCR products from Figures 5 and 6 were calculated with the ImageJ software. Click here for file

Additional File 3 Graphic quantification of the relative viral abundance in SupT1. The density of the PCR products from Additional file 1 were calculated with the ImageJ software. Click here for file
