# Supplementary material for: Trans-inhibition of HIV-1 by a long hairpin RNA expressed within the viral genome
Source: Retrovirology. 2007 Mar 1;4:15. doi: 10.1186/1742-4690-4-15 (PMC1819390; doi:10.1186/1742-4690-4-15)
Supplement: Additional File 1 — Virus competition experiments in SupT1 and PBMC. The composition of the wild type HIV-1 and the AS escape mutants was followed by PCR across the Nef region with primers tTA1 and CN1 (see Fig. 1). [file 1742-4690-4-15-S1.ppt]

## Slide 1
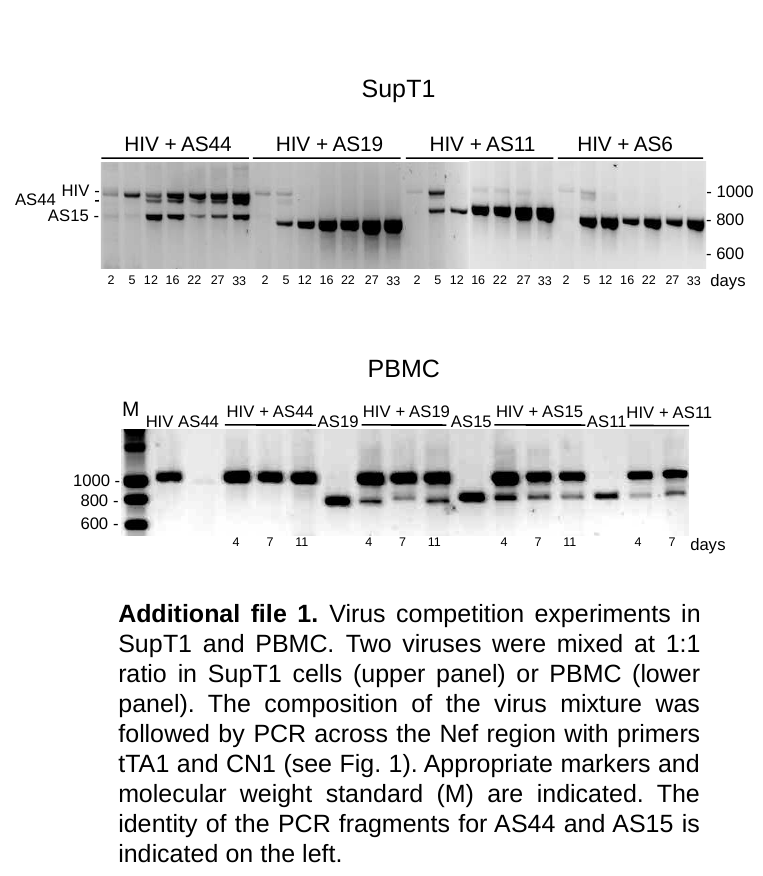

SupT1
HIV + AS44
HIV + AS19
HIV + AS11
HIV + AS6
HIV -
- 1000
AS44
AS15 -
- 800
- 600
days
2
5
12
16
22
27
33
2
5
12
16
22
27
33
2
5
12
16
22
27
33
2
5
12
16
22
27
33
PBMC
M
HIV + AS44
HIV + AS19
HIV + AS15
HIV + AS11
HIV
AS19
AS15
AS44
AS11
 1000 -
800 -
 600 -
4
7
11
4
7
11
4
7
11
4
7
days
Additional file 1. Virus competition experiments in SupT1 and PBMC. Two viruses were mixed at 1:1 ratio in SupT1 cells (upper panel) or PBMC (lower panel). The composition of the virus mixture was followed by PCR across the Nef region with primers tTA1 and CN1 (see Fig. 1). Appropriate markers and molecular weight standard (M) are indicated. The identity of the PCR fragments for AS44 and AS15 is indicated on the left.
