# Supplementary material for: Trans-inhibition of HIV-1 by a long hairpin RNA expressed within the viral genome
Source: Retrovirology. 2007 Mar 1;4:15. doi: 10.1186/1742-4690-4-15 (PMC1819390; doi:10.1186/1742-4690-4-15)
Supplement: Additional File 2 — Graphic quantification of the relative viral abundance in SupT1 and PBMC. The density of the PCR products from Figures 5 and 6 were calculated with the ImageJ software. [file 1742-4690-4-15-S2.ppt]

## Slide 1
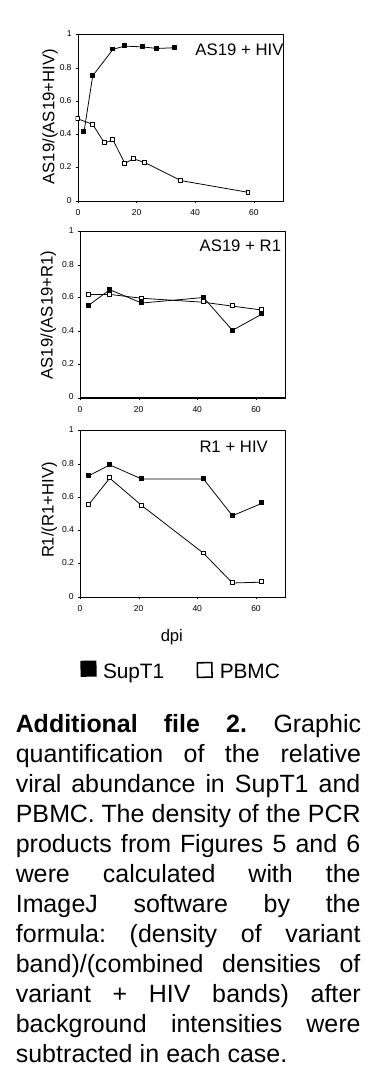

AS19 + HIV
AS19/(AS19+HIV)
AS19 + R1
AS19/(AS19+R1)
R1 + HIV
R1/(R1+HIV)
dpi
SupT1
PBMC
Additional file 2. Graphic quantification of the relative viral abundance in SupT1 and PBMC. The density of the PCR products from Figures 5 and 6 were calculated with the ImageJ software by the formula: (density of variant band)/(combined densities of variant + HIV bands) after background intensities were subtracted in each case.
