# Supplementary material for: Trans-inhibition of HIV-1 by a long hairpin RNA expressed within the viral genome
Source: Retrovirology. 2007 Mar 1;4:15. doi: 10.1186/1742-4690-4-15 (PMC1819390; doi:10.1186/1742-4690-4-15)
Supplement: Additional File 3 — Graphic quantification of the relative viral abundance in SupT1. The density of the PCR products from Additional file 1 were calculated with the ImageJ software. [file 1742-4690-4-15-S3.ppt]

## Slide 1
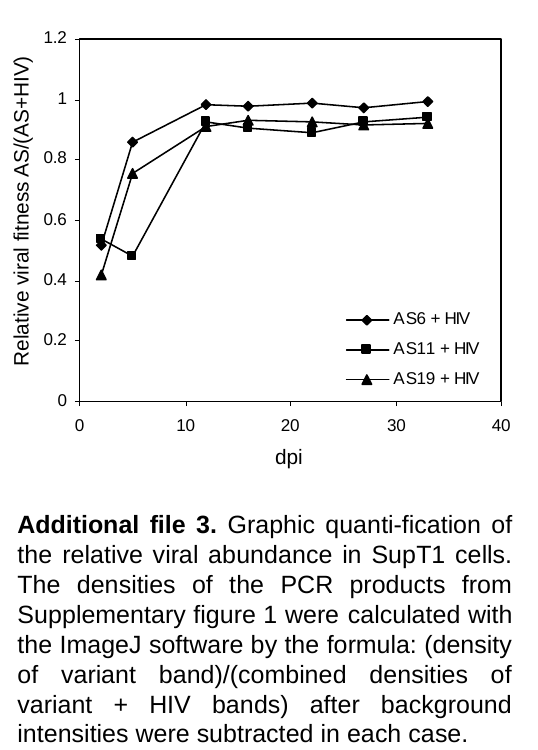

Relative viral fitness AS/(AS+HIV)
dpi
Additional file 3. Graphic quanti-fication of the relative viral abundance in SupT1 cells. The densities of the PCR products from Supplementary figure 1 were calculated with the ImageJ software by the formula: (density of variant band)/(combined densities of variant + HIV bands) after background intensities were subtracted in each case.
